# Eicosanoids: Exploiting Insect Immunity to Improve Biological Control Programs

**DOI:** 10.3390/insects3020492

**Published:** 2012-05-16

**Authors:** David Stanley, Eric Haas, Jon Miller

**Affiliations:** 1 Biological Control of Insects Research Laboratory, USDA Agricultural Research Service, 1503 S. Providence Rd., Columbia, MO 65203, USA; 2 Department of Chemistry, Creighton University, Omaha, NE 68178, USA; E-Mail: erichaas@creighton.edu; 3 Department of Biological Sciences, Northern Illinois University, DeKalb, IL 60115, USA; E-Mail: jsmiller@niu.edu

**Keywords:** biological control, insect immunity, signal transduction, eicosanoids, prostaglandins, phospholipase A_2_

## Abstract

Insects, like all invertebrates, express robust innate, but not adaptive, immune reactions to infection and invasion. Insect immunity is usually resolved into three major components. The integument serves as a physical barrier to infections. Within the hemocoel, the circulating hemocytes are the temporal first line of defense, responsible for clearing the majority of infecting bacterial cells from circulation. Specific cellular defenses include phagocytosis, microaggregation of hemocytes with adhering bacteria, nodulation and encapsulation. Infections also stimulate the humoral component of immunity, which involves the induced expression of genes encoding antimicrobial peptides and activation of prophenoloxidase. These peptides appear in the hemolymph of challenged insects 6–12 hours after the challenge. Prostaglandins and other eicosanoids are crucial mediators of innate immune responses. Eicosanoid biosynthesis is stimulated by infection in insects. Inhibition of eicosanoid biosynthesis lethally renders experimental insects unable to clear bacterial infection from hemolymph. Eicosanoids mediate specific cell actions, including phagocytosis, microaggregation, nodulation, hemocyte migration, hemocyte spreading and the release of prophenoloxidase from oenocytoids. Some invaders have evolved mechanisms to suppress insect immunity; a few of them suppress immunity by targeting the first step in the eicosanoid biosynthesis pathways, the enzyme phospholipase A_2_. We proposed research designed to cripple insect immunity as a technology to improve biological control of insects. We used dsRNA to silence insect genes encoding phospholipase A_2_, and thereby inhibited the nodulation reaction to infection. The purpose of this article is to place our view of applying dsRNA technologies into the context of eicosanoid actions in insect immunity. The long-term significance of research in this area lies in developing new pest management technologies to contribute to food security in a world with a rapidly growing human population.

## 1. Introduction

Insects and other invertebrates have been used a model systems since of the beginning of immunological research. Edward Jenner, an English country doctor, is credited with conducting the first experiment in immunology, in the 1790’s. He exposed a man to cowpox, and then injected him with smallpox; the human “model” became ill, but quickly recovered. During his work on cholera in fowls nearly 100 years later, Louis Pasteur discovered what are now called attenuated vaccines in the 1870’s. Elie Metchnikoff, born in Kharkov, Russia, was an embryologist who became fascinated with what he called phagocytes. He studied phagocytes, first as an embryological study; his seminal observation with respect to animal immunology came in 1882, after he moved from Russia to Messina, Italy. He placed a splinter into the transparent body of a starfish, the bipinnaria larval stage, then observed phagocytes surrounding the splinter. This observation broadened Metchnikoff’s vision of phagocytes from “eating to feed” to “eating to defend” [1]. Metchnikoff shared the Nobel Prize with Paul Ehrlich in 1908 “in recognition of their work on immunity”. He also studied other invertebrates, particularly water fleas. The Russian Serguei Metalnikov worked in Metchnikoff’s laboratory (in Italy) for a couple of years, returned to Russia and finally moved to the Pasteur Institute in 1919, where he stayed for the rest of his life. Metalnikov worked on insect immunity in the 1920’s, using a variety of insects, although larvae of the greater wax moth, *Gallaria mellonella,* were his main model animal [2]. Metalnikov was one of the founders of insect immunology, recognized by Bulet and his colleagues by naming a series of insect antibacterial peptides “metalnikowins” [3]. 

Insect immunology is by now a mature field, with a very large body of literature and many active scientists researching diverse aspects of the field. Although it is commonly noted that insects lack the antibody-based adaptive immune systems known in vertebrates, insect immunity is a highly effective protection system. Insect immunity is exclusively innate immunity, that is, a naturally occurring, non-specific immunity that does not depend on previous infection experience. Aside from direct studies of immune functions, study of innate immunity in the absence of adaptive immunity informs research into the evolution of animal immune systems. Immune functions are biologically expensive and studies of ecological immunity are revealing physiological trade-offs, in which expensive immunity are traded off for other biologically expensive functions, including reproduction and migrations [4]. A recent field study [5] documents the importance of insect immunity in nature, showing that virtually all of insect specimens collected from agrarian fields had been infected and had recovered from the infectious events. All this research highlights the breadth and depth of insect immunology, which is now beyond the capacity of individuals or even groups to comprehensively treat. Prostaglandins (PGs) and other eicosanoids are crucial mediators of insect immunity. Inhibition of eicosanoid biosynthesis lethally impairs insect immune reactions to infection, although at the time the eicosanoid actions were discovered there was no realistic vision of how eicosanoid signaling could contribute to new pest control technologies. However, the recent advent of gene silencing tools opens the possibility of applying knowledge of eicosanoid signaling to insect pest control technologies. In this brief paper we sketch insect immunity, outline eicosanoid systems and review the roles of eicosanoids as crucial mediators of insect immune functions. Insect immunity exerts sufficient selective force on some pathogens and parasitoids to drive evolution of mechanisms to suppress host immunity and we highlight a couple of examples. Finally, we report on our efforts to cripple pest insect immunity using molecular tools.

## 2. A Sketch of Insect Immunity

**Figure 1:**
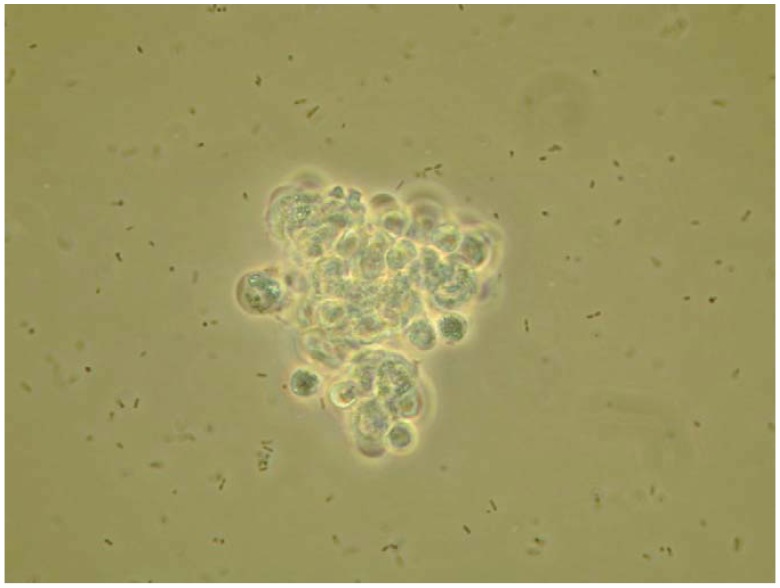
A microaggregate of hemocytes (approximately 10–12 μm) formed at 1 h after injecting *S. marcescens* into the hemocoel of a tobacco hornworm. For this microphotograph (taken 1 h after injection), hemolymph was withdrawn, diluted with buffer and placed on a microscope slide for observation and photography. The cells in these photographs range from 10–12 microns. Photo by JSM.

**Figure 2:**
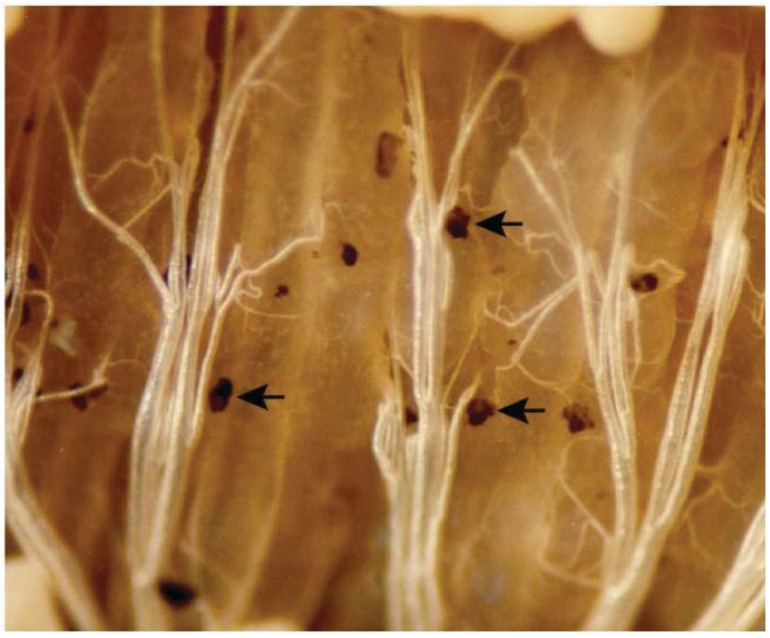
Nodules formed 4 h following an artificial infection by injecting the bacterium *Serratia marcescens*, into the hemocoel of a tobacco hornworm. The arrows point to melanotic nodules (approximately 0.1 mm in diameter) against the background of the highly tracheated midgut.

Insect immunity is traditionally resolved into three main components. The integument and possibly the peritrophic membrane, act as a physical barrier to infection. Once these barriers are breached, cellular (or hemocytic) defenses make up the immediate, second line of defense. Some of the biochemical signaling responsible for launching insect cellular immune reactions can be detected within seconds following infection. Cellular defenses include phagocytosis and encapsulation. Phagocytosis is the cellular internalization of bacterial cells or fungal spores, which are secondarily killed within hemocytes. Parasitoid eggs and other invaders that are too large for phagocytosis are encapsulated by circulating hemocytes. Nodulation is a form of encapsulation in which microaggregations of hemocytes (shown in Figure 1) with adhering bacterial cells grow into large nodules (Figure 2). The nodules are completed with a surrounding layer of plasmatocytes that express an active phenoloxidase (PO) that melanizes the nodules. The melanization process produces reactive oxygen forms that may chemically kill the adhering microbes. This process is responsible for removing the vast majority of infecting bacterial cells from hemolymph circulation [6]. Nodules are attached to an inner body wall or an organ, where they remain for the life of the insect. 

Humoral immunity refers to the induced biosynthesis of antimicrobial peptides and proteins (AMPs). Cecropin is the classical antimicrobial peptide, so named from pupae of the Cecropia moth, *Hyalophora cecropia* from which it was originally isolated [7]. In the subsequent years hundreds of animal AMPs have been isolated and their structures determined [8]. Activation of the genes encoding these many peptides in *Drosophila* begins with sensing microbes within the body. Sensing microbial infections leads to activation of the Toll and Imd pathways, which ultimately leads to induced synthesis of the peptides. The detailed picture is beyond the scope of this article and can be accessed through recent reviews [9]. Antimicrobial peptides appear in hemolymph circulation hours after an infection occurs and Haine *et al*. [6] suggested these peptides serve a secondary, “mop up” role in containing infections. 

Antimicrobial peptides also serve a crucial role in prophylactic immunity. An *et al*. [10] report that homodimers of the tanning neuropeptide, bursicon (which acts in the heterodimer configuration), induce expression of genes encoding antimicrobial peptides during the vulnerable molting cycle from pupae to adult flies, *Drosophila melanogaster*. They showed the homodimers stimulate >19-fold increase in expression of genes encoding eight antibacterial peptide genes, the homodimers do not act through the bursicon heterodimer receptor, and they activate the Imd pathway via the transcription factor Relish. 

Of course, humoral and cellular immune functions overlap. Prophenoloxidase, for example, is released from oenocytoids, one type of hemocyte. Prostaglandins (PGs) and other eicosanoids are crucial biochemical signals responsible for mediating and coordinating insect cellular immune reactions to infection. We now turn to an outline of eicosanoid systems.

## 3. An Outline of Eicosanoid Biosynthesis and Action

Our appreciation of eicosanoids dates to the early 1930’s, when von Euler [11] reported that a lipoidal, acidic substance produced in the prostate gland stimulated contractions in uterine smooth muscle preparations. He named these substances PGs, although the structures of these compounds were not determined until nearly 30 years later [12]. Vane’s discovery, that asprin and other non-steroidal anti-inflammatory drugs act by inhibiting PG biosynthesis [13], launched a tremendous research enterprise into the chemistry, biology, pharmacology and molecular biology of PGs and other eicosanoids that continues to gain momentum. Bergstrom, his student Samuelsson and Vane shared the 1982 Nobel Prize in Physiology or Medicine “for their discoveries concerning prostaglandins and related biologically active substances”. The related biologically active substances include thromboxanes, hydroxyeicosatrienoic acids, leukotrienes and lipoxins to mention a few. Corey *et al*. [14] coined the term eicosanoid, from the Greek word “eikosi”, meaning twenty. Eicosanoid is a broad term for all biologically active, oxygenated metabolites of arachidonic acid (AA; 20:4n-6) and two other C20 polyunsaturated fatty acids. Corey also accepted a Nobel Prize (1990) for his work in developing strategies in organic synthesis, including eicosanoids and several other classes of chemicals.

Our understanding of eicosanoids comes from research in universities, public and private sectors on the biomedical significance of these compounds in human and veterinary medicine. This work has generated a very large body of information which, for convenience we refer to as the mammalian model. The mammalian model is useful in generating hypotheses, developing reagents and protocols for research and for understanding results. The model is also misleading, as we will see.

**Figure 3:**
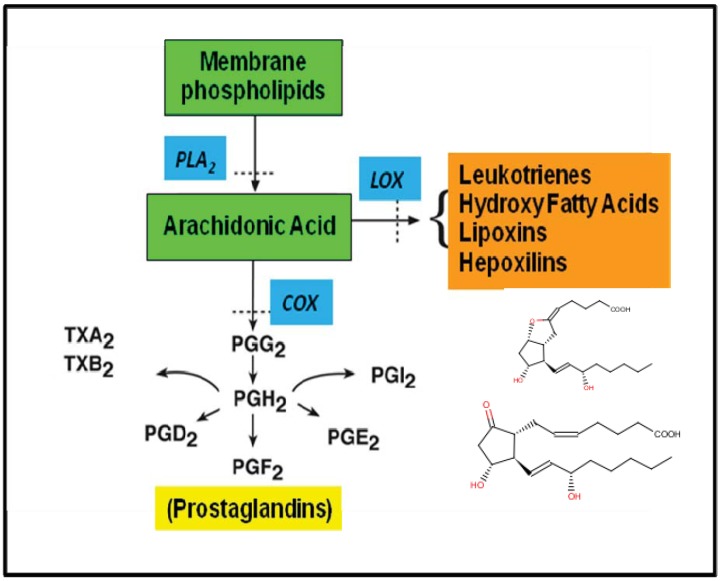
An outline of eicosanoid biosynthesis. Here, arachidonic acid (AA) is liberated from the membrane phospholipids via activation of a phospholipase A_2_. The free fatty acids are subsequently metabolized via two enzymic pathways: (i) The cyclooxygenase (COX) pathway, forming prostaglandins, thromboxanes or prostacyclins; and (ii) the various lipoxygenase (LOX) pathways, forming leukotrienes, lipoxins, hepoxilins and hydro(pero)xy- and hydroxy-fatty acids. The blue boxes indicate enzymes that are inhibited by various pharmaceutical products.

Eicosanoid biosynthesis is outlined in Figure 3. Phospholipase A_2_ (PLA_2_) catalyzes the first step in the process, the hydrolysis of AA from cellular phospholipid pools. While 15 PLA_2_ groups are recognized [15], it is convenient to recognize secretory and cellular enzymes (sPLA_2_ and cPLA_2_). sPLA_2_s include those in reptile, bee and wasp venoms, digestive sPLA_2_s and some PLA_2_s that act in pathophysiology, such as osteoarthritis. These are typically low molecular weight, globular proteins with multiple disulfide bridges. cPLA_2_s are larger proteins, up to 115 kDa, that act in specific cellular functions, such as phospholipid remodeling. Some PLA_2_s in both broad groups act in eicosanoid biosynthesis. Depending on the cell-specific complement of enzymes, the free AA is substrate for cyclooxygenases (COXs) and lipoxygenases (LOXs). The COX protein contains two enzyme activities, a COX that converts AA into PGG_2_ and a hydroperoxidase that converts PGG_2_ into PGH_2_. Again depending on the cell-specific complement of enzymes, PGH_2_ can be converted into PGE_2_ by an isomerase, into PGD_2_ by another isomerase, into PGF_2__α_ by a reductase, into PGI_2_ (also called prostacyclin) by prostacyclin synthase or into thromboxane B_2_ by thromboxane synthase. Two forms of COX are recognized in mammalian cells, a COX-1 and a COX-2. COX-1 is a constitutive enzyme responsible for cellular housekeeping actions, such as ion transport physiology. COX-2 is an inducible enzyme that produces PGs in response to emergencies, such as injury, infection and mitogens. 

Mammalian cells express several LOXs that convert AA into a wide range of compounds, including hydroperoxyeicosatetraenoic acids, hydroxyeicosatetraenoic acids, leukotrienes, hepoxilins, trioxilins and lipoxins. These compounds mediate many physiological and pathophysiological events. A mixture of leukotrienes, for example, makes up the slow reacting substance of anaphylaxis. Mammalian cells also produce epoxyeicosatrienoic acids; however, these compounds have not yet been reported for invertebrates. 

Eicosanoid structures and biosynthetic pathways are detailed elsewhere [16,17].

## 4. Eicosanoids Are Crucial Mediators of Insect Cellular Immunity

Although some of the earliest research into immunology was conducted with invertebrates, research into insect immunology lost momentum after the 1920’s as attention shifted from innate to adaptive immunity. The isolation of inducible antibacterial proteins (later named cecropins) from hemolymph of infected *Hyalophora cecropia* pupae [18,19] opened a new range of insect immunology and focused interest on insect humoral immunity. By the late 1980’s more inducible antimicrobial peptides were identified. Because these peptides were inducible, they became a useful model for work on gene regulation. Work in this area led to discovery of the immune deficiency [20] pathway and to understanding that the Toll pathway, first known in developmental genetics, also regulates expression of immune genes. In this context, Stanley-Samuelson and his colleagues investigated the biochemical signals responsible for mediating insect hemocytic immunity. They found that treating tobacco hormworms with pharmaceutical inhibitors of eicosanoid biosynthesis disabled clearance of injected bacterial from hemolymph circulation [21]. Because all experiments were conducted within 2–4 h post-infection, they speculated that eicosanoids mediated cellular, as opposed to humoral, immune reactions. This was a reasonable speculation and in a second series of experiments, they demonstrated that two hemocytic immune functions, microaggregation and nodulation, are mediated by eicosanoids [22].

The subsequent research took two separate pathways. In one, the biochemistry of PG biosynthesis in tobacco hornworm fat body and hemocytes, the insect immunity-conferring tissues was characterized. This work showed that fat body is competent to produce PGs, PGA_2_ is the major product and PG biosynthesis was inhibited in reactions conducted in the presence of known pharmaceutical non-steroidal anti-inflammatory drugs (NSAIDs) [23]. In mammals PGA_2_ is produced from a spontaneous dehydration of PGE_2_, not by a direct enzymatic step. Insects mark an important departure from the mammalian model because PGE_2_ is not rearranged into PGA_2_ in the hornworm preparations, from which it was suggested that PGA_2_ is formed by way of an unidentified intermediate. Tobacco hornworm hemocytes also are competent to produce eicosanoids, however, the major hemocyte product was a LOX product, then tentatively identified as a hydroxyeicosatetraenoic acid [24]. This work documented eicosanoid biosynthesis in an insect species.

As shown in Figure 3, PLA_2_ is the first step in eicosanoid biosynthesis. Early experiments with eicosanoids showed that inhibition of PLA_2_ activity severely impair insect nodulation reactions, from which it was inferred that PLA_2_ also is a critical step in eicosanoid biosynthesis in insect tissues. Stanley-Samuelson and his colleagues recorded a calcium-dependent PLA_2_ in hornworm fat body preparations [25] and a calcium-inhibited PLA_2_ in hemocyte preparations [26]. Figueiredo *et al*. [27] also reported that inhibition of PLA_2_ reduced phagocytosis reactions in the blood-sucking bug, *Rhodnius prolixus*. This and other work indicates the crucial role of PLA_2_s in insect immunity [28]. 

Aside from the work on eicosanoid biosynthesis, several laboratories tested the idea that eicosanoids mediate insect immune reactions to infection or invasion, updated from Stanley and Miller ([29], Table 1). The table shows that eicosanoids mediate cellular immune reactions to challenge in 29 insect species representing seven Orders. Bacteria, fungi, viruses, a parasitoid and a protozoan, as well as bacterial components and glass beads stimulate eicosanoid-mediated immune reactions. This summary supports the statement that eicosanoids mediate insect cellular immune reactions to infection and invasion generally. From here it is a small step toward research into specific eicosanoid actions in cellular immunity.

**Table 1:** Eicosanoids mediate cellular immune reactions to immune challenge in juvenile and adult representatives of seven insect orders.

Species	Life stage	Immune elicitor	Reference
**Lepidoptera**			
*Manduca sexta*	larvae	*Serratia marcescens*	[22]
		*Beauveria bassiana*	[63]
		*Metarhizium anisopliae*	[58]
*Agrotis ipsilon*	larvae	*S. marcescens*	[77]
*P. unipuncta*	larvae	*S. marcescens*	[77]
*G. mellonella*	larvae	glass beads	[30]
*Bombyx mori*	larvae	*S. marcescens*	[71]
*Colias eurytheme*	larvae	*S. marcescens*	[70]
*Spodoptera exigua *	larvae	*Xenorhabdus nematophila*	[44]
		BAWNPV	[69]
*S. frugiperda*	larvae	SfNPV	[69]
*Ostrinia nubilalis*	larvae	*S. marcescens*	[74]
*Galleria mellonella *	larvae	Virus	[55]
*Pieris brassicae *	larvae	*B. bassiana*	[72]
*Lymantria dispar*	larvae	LdMNPV	[68]
*Helicoverpa zea*	larvae	HzSNPV	[69]
**Coleoptera**			
*Zophobas attraus*	larvae	*S. marcesens*	[64]
		Lipopolysaccharide	[54]
*Tribolium castaneum*	larvae	*E. coli*	[51]
**Diptera**			
*D. melanogaster*	larvae	*L. boulardi* eggs	[56]
*Neobellieria bullata*	larvae	laminarin	[60]
*Anopheles albimanus*	adult	*Micrococcus luteus*	[61]
		*Klebsiella pneumonia*	[61]
*Chryusomya megacephala*	larvae	*Ureaplasma urealyticum*	[76]
**Hymenoptera**			
*Apis mellifera*	adult	*S. marcescens*	[67]
*Pimpla turionellae*	adult	Herpes virus	[59]
**Orthoptera**			
*Gryllus assimilis*	adult	*S. marcesens*	[65]
*G. firmus*	adult	*X. nematophila*	[66]
*P. americana *	adult	*S. marcesens*	[73]
*L. migratoria*	adult	laminarin	[62]
**Homoptera**			
*M. septendecim*	adult	*S. marcesens*	[75]
*M. cassini*	adult	*S.marcesens*	[75]
*Dactylopius coccus *	adult	laminarin	[57]
**Hemiptera**			
*R. prolixus*	larvae	*T. rangeli*	[47]

## 5. Eicosanoids Influence Specific Cellular Actions

**Figure 4:**
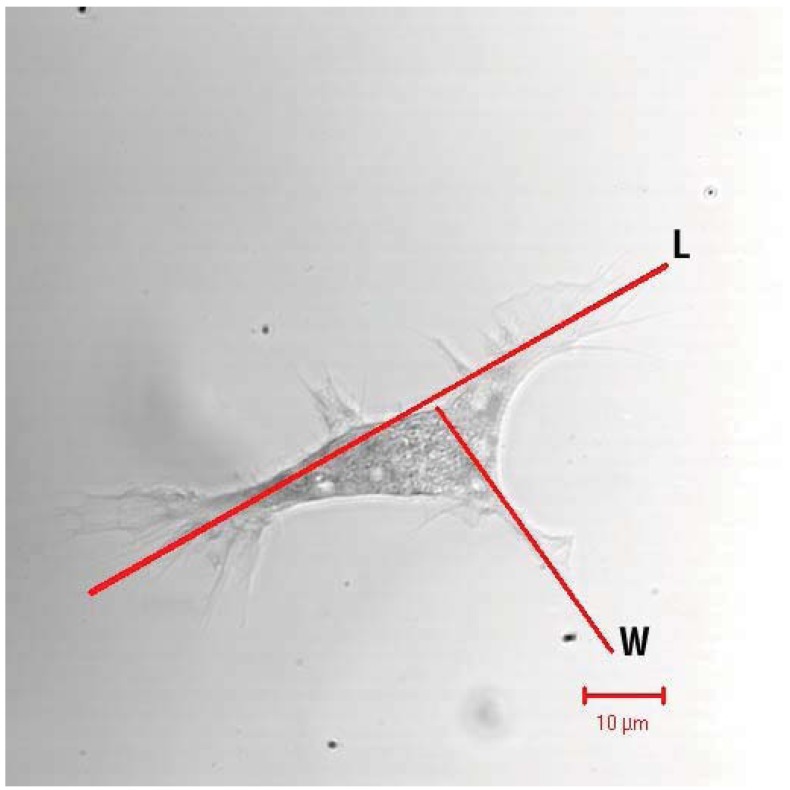
Eicosanoids act in plasmatocytes elongation on glass slides. The line indicates the method of measuring the length (marked L) and the corresponding width (marked W). Photo by JSM.

The pioneering work on the roles of eicosanoids in insect immunity revealed that pharmaceutical inhibitors of eicosanoid biosynthesis lethally impaired clearance of injected bacteria from hemolymph circulation [21], with only speculation on what the eicosanoids do. Miller *et al*. [22] showed that eicosanoids mediate microaggregation and nodulation reactions to bacterial infection (Figure 1 and Figure 2). Mandato *et al*. [30] added to the list of eicosanoid-mediated actions, showing that eicosanoids act in prophenoloxidase (PPO) activation, phagocytosis and cell spreading in larval *Galleria mellonella*. Figueiredo *et al*. [27] also showed that inhibiting PLA_2_ substantially reduced phagocytosis of the parasitic protozoan *Typanosoma rangeli* by *R. prolixus* hemocytes and that the inhibition was reversed by treating *R. prolixus* with AA. They concluded that eicosanoids mediate phagocytosis in *Rhodnius*. Similarly, Miller [31] studied tobacco hornworm plasmatocytes to confirm that eicosanoids act in cell spreading (Figure 4). Hemocyte adhesion is an important action in clearing infecting bacteria from circulation. Marin *et al*. [32] reported that AA increased adhesion of *G. mellonella* granulocytes, but not plasmatocytes, to a glass surface. More recently, Merchant *et al*. [33] showed that eicosanoids influence tobacco hornworm hemocyte migration in Boyden chambers. This may be a specific PG function because a LOX inhibitor, esculetin, did not influence hemocyte migration. Finally, Shrestha and Kim [34] discovered that eicosanoids mediate release of PPO from oenocytoids by inducing cell lysis. These visible eicosanoid-mediated actions are the culminations of an unknown number of unseen, intracellular events. Together, they indicate the potent biological significance of eicosanoids in insect immune functions.

**Figure 5:**
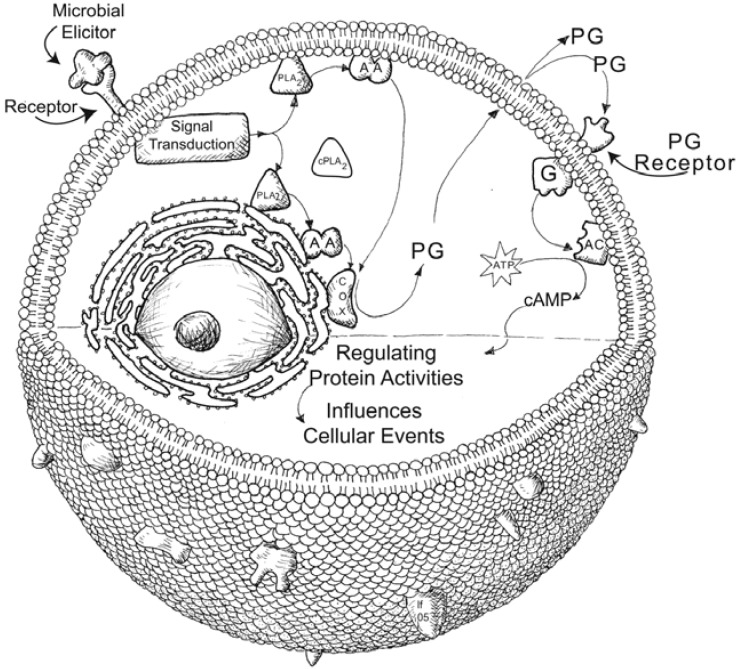
A hemocyte model depicting a general mechanism of Prostaglandin (PG) signal transduction. After stimulating a pattern receptor, an intracellular PLA_2_ is activated to hydrolyze AA from cellular phospholipids. The free fatty acid is subsequently oxygenated into a PG or other eicosanoids. For PGs, specific transporter proteins export them out the cell. The exported PGs can bind to receptors located on the cell surface in an autocrine action or can interact with other hemocytes in a paracrine action. We image that both mechanisms operate in mediating hemocyte immune reactions.

Eicosanoids act in several distinct cellular defense functions, including phagocytosis, microaggregation, nodulation, adhesion, hemocyte migration, plasmatocyte elongation and release of PPO from oenocytoids. These findings prompt the issue of eicosanoid modes of action in insect hemocytes. Figure 5 shows a general model of how PGs exert their actions in cells, drawn from the mammalian model. In the model hemocyte interaction with a chemical component of an invader (perhaps lipopolysaccharide from Gram-negative bacteria) activates a PLA_2_, which ultimately leads to PG biosynthesis. The PGs are exported from the cell, where they subsequently interact with specific G-protein coupled receptors (GPCRs) on the cell that produced the PGs (autocrine action) or on nearby cells (paracrine action). The mammalian model provides a very deep background on PG receptors; however, we have very little information on PG action modes in insect cells. In their work on hemocyte adhesion, Marin *et al*. [32] reported that cAMP influences hemocytes. With respect to eicosanoids, as just mentioned, AA influenced hemocyte adhesion, however, neither AA nor PGE_2_ influenced the intracellular concentrations of cAMP. While cAMP is certainly involved in hemocyte actions, the influence of AA on cell adhesion is exerted through a signal mechanism other than cAMP. 

Shrestha and Kim [34] suggested that eicosanoid-mediated oenocytoid lysis involves the protein kinase C (PKC) pathway. This idea is based on their finding that specific PKC inhibitors inhibited oenocytoid lysis. Using other inhibitors, they produced a hypothetical model in which a PG acts through a GPCR to activate a phospholipase C, which may release inositol-3-phosphate from cellular phospholipids and thereby release calcium from intracellular compartments to activate PKC. They went on to clone and identify a specific PGE_2_ receptor in oenocytoids and show the receptor is physiologically active [35].

Stanley *et al*. [36] suggested another mode of PG action, showing that PGs influence protein expression in an established insect cell line. While protein expression can be modulated at several levels, we also showed that for some proteins, the change in protein expression was parallel to changes in mRNA levels. It follows that PGs may influence gene expression for some of the proteins. We continue working on the influence of PGs on protein expression, finding that PGA_2_, the major PG product in our characterization of PG biosynthesis in hornworm fat body, influences the expression of many more proteins than the other PGs [37]. These few reports highlight the scarcity of information on PG actions in insect immunology and insect systems generally. This remains an open frontier in insect science.

## 6. Eicosanoids Influence Humoral Immune Reactions

Morishima *et al*. [38] first reported that eicosanoids act in up-regulation of immune genes. Using whole larvae and fat body isolated from the silkworm, *Bombyx mori*, they showed that soluble peptioglycan stimulated expression of genes encoding Cecropin B and lysozyme. The stimulation was inhibited by treating the experimental preparations with inhibitors of PLA_2_, cyclooxygenase or lipoxygenases. They also showed that AA treatments led to induction of the cecropin and lysozyme genes, from which they inferred that eicosanoids act in expression of at least these two immune genes following recognition of peptidoglycan. 

In a different line of experimentation, Yajima *et al*. [39], developed a screening system based on activating the imd pathway in *Drosophila*. They found that inhibitors of PLA_2_, the first step in eicosanoid biosynthesis, blocked lipopolysaccharide-dependent activation of the immune deficiency pathway. The blockage was reversed by AA treatments. Because AA did not independently stimulate the imd pathway, the authors concluded that eicosanoids participate in imd activation, but require additional signaling.

Shrestha and Kim [40] reported that treating beet armyworms, *Spodoptera exigua*, with injected cocktails of dexamethasone (Dex; a glucocorticoid PLA_2_ inhibitor) and *Escherichia coli* Top10 bacteria led to dose-dependent decreases in expression of genes encoding a range of immune-conferring proteins, including Cecropin A, lysozyme, prophenoloxidase, serpin 2 and dopa decarboxylase. The inhibitory influence of Dex was reversed in *S. exigua* larvae treated with the Dex/bacteria cocktail plus various individual eicosanoids. They found PGA_1_, PGD_2_, PGE_2_, PGF_2__α_ and leukotriene B_4_ completely reversed the Dex effect, while PGB_2_ and PGE_1_ reversed the effect for all but the gene encoding prophenoloxidase. It is not clear how seven individual eicosanoids all act in reversing the influence of Dex on gene expression. Nonetheless, there is increasing evidence that eicosanoids act in humoral, as well as cellular, immune reactions to microbial infections. 

Eicosanoids also mediate the actions of an insect cytokine, plasmatocyte spreading peptide (PSP). Srikanth *et al*. [41] reported that PSP and PGE_2_ independently induced plasmatocyte spreading, that inhibitors of PG biosynthesis reversibly inhibited plasmatocyte spreading, that silencing the gene encoding pro-PSP thwarted plasmatocyte spreading and that inhibitors of PG biosynthesis impaired PSP-stimulated plasmatocyte spreading. The authors offered a model showing that PSP influences cell spreading via stimulating eicosanoid biosynthesis. 

## 7. Invaders Target Eicosanoid Biosynthesis at the PLA_2_ Step to Suppress Host Immunity

An underlying theme of this article is that insects have evolved a robust arsenal of immune defenses to stave off infections and invasions. In the Introduction we commented on a field study designed to assess the hypothesis that insects commonly experience infections in nature [5]. The point of the study is that, indeed, insects experience infections and invasions in nature, including agrarian fields. More important, however, is that they overcome the infections. It is not surprising, seen from the perspective of insect immunity, that invaders have evolved mechanisms to avoid or suppress the immune systems of their host insects. For a single example, Fang *et al*. [42] reported that immune response genes of a lepidopteran host, *Pieris rapae*, are suppressed by venom of the parasitoid *Pteromalus puparum*. The range of parasitoid strategies to avoid or suppress host immunity is very well treated in the now classic review by Strand and Pech [43]. 

Some insect pathogenic bacteria suppress host immune reactions by targeting a key component of the eicosanoid signaling system, PLA_2_, again, the first step in eicosanoid biosynthesis. The entomopathic nematode, *Steinernema carpocapasae*, lives in a mutualistic relationship with the bacterium, *Xenorhabdus nematophila*. After entering an insect body, the nematode voids *X. nematophila* into the hemolymph. The bacterium rapidly increases in population size and kills the insect host. The killing probably serves the mutualistic nematode partner in two ways. First, the freshly killed insect provides the nematode with an appropriate microhabitat to complete development and reproduce. Second, *X. nematophil*a protects the nematode by crippling the host immune system. Working with larvae of the beet armyworm, *S. exigua*, Park and Kim [44] first suggested that the bacterium impairs host immunity by inhibiting eicosanoid biosynthesis. This was based on their finding that treating the host with AA reduced larval mortality by about 40%. Additional research showed that *X. nematophila* produces and secretes factors that directly inhibit sPLA_2_ in the host. The bacterial factors are potent sPLA_2_ inhibitors: they inhibit sPLA_2_s from insects, prokaryotic and vertebrate sources [45]. Kim *et al*. [46] showed that a related bacterium, *Photorhabdus temperata* (also a mutulistic partner of nematodes) similarly inhibits PLA_2_ and nodulation in its host, *S. exigua*. The authors suggested that bacteria in the genera *Xenorhabdus* and *Photorhabdus* generally share the ability to inhibit PLA_2_ in their hosts.

The strategy of impairing host immunity via compromising eicosanoid biosynthesis is not limited to bacteria. Garcia *et al*. [47] reported that after feeding 5^th^-instar larvae of *R. prolixus* on blood containing juveniles of the protozoan *T. rangeli*, injecting additional *T. rangeli* results in reduced hemocyte aggregation and increased mortality. These effects were reversed when the protozoans were co-injected into the host with AA (at 10 μg/insect). The authors suggested that oral infection with *T. rangeli* inhibits the release of AA for eicosanoid biosynthesis. They later showed that adding the glucocorticoid, Dexamethasone, to the blood meals of *R. prolixus* inhibited phagocytosis and the inhibition was reversed by treating experimental *R. proxilus* larvae with AA or, separately, with platelet activating factor, reversed the dexamethasone inhibition [27]. In a second series of experiments they showed that *T. rangeli* cells inhibit phagocytosis and the inhibition was reversed by AA treatments. Then using a fluorimetric assay, they demonstrated that *T. rangeli* cells inhibit PLA_2_ activity in *R. prolixus* hemocytes. This work shows that the protozoan parasite suppresses a host immune function, phagocytosis, by targeting the PLA_2_ components of eicosanoid signaling. 

There also is a larger message in these findings. Insect invaders have evolved many strategies to avoid or suppress insect immunity, most of which are outside the scope of this paper. These strategies emerged from the tremendous selection forces on invaders. The fact that some of the invaders operate by inhibiting a key enzyme in eicosanoid signaling is a convincing argument that eicosanoids are crucial mediators of insect immunity. This brings us to the final section of this paper.

## 8. Improving Biological Control Technologies: Targeting Insect Eicosanoid Signaling

Oerke and Dehne [48] estimated global crop losses to pests at about 30–40% per year, depending on the particular crop, a large proportion of which is due to insects. In U.S., total crop losses to pathogens, weeds and insects is about 33% (13% due to insects) of potential crop value. More important, losses are increasing, despite advances in pest management technologies and programs [49]. Much of the increased potentials for insect crop damage are tied to climate change. Global warming is expected to favor several important aspects of insect biology, including growth rates, numbers of generations per year and geographic ranges. While crop losses to pests increase, projections of human population growth indicate steady increases from the current 6.8 billion to as many as 9.3 billion people by 2050 (http://esa.un.org/unpd/wpp/index.htm). As major competitors for human food, insects represent a growing threat to world food security.

Despite their threatening posture, the development of new technologies and tools to manage pest insect populations is seriously lagging. Thirty-seven years ago, Djerassi *et al*. [50] recognized the need for new technologies in insect control and the increasing time required to convert new discoveries into practical applications. We predict that long-term sustainable improvements in agricultural production will depend on continuous invention of new pest management systems and discovery of important enhancements of existing systems. Developing practical protocols for targeting insect immunity directly is not novel as a concept, as can be seen in virtually every grant proposal on insect immunology. Nonetheless, we now have molecular tools that may help translate the concept into applicable research, specifically, molecular constructs to silence immunity-related genes may lead to several different avenues of attacking pest insects. 

PLA_2_ is the major point of attack evolved in the bacteria and protozoan discussed just above. Because of its position in the eicosanoid biosynthetic pathways, this enzyme is a key vulnerability in insect immunology. Shrestha *et al*. [51] demonstrated the concept of impairing insect immunity by silencing genes encoding sPLA_2_s. We showed that challenging late instar *Tribolium castaneum* with the bacterium *E. coli* evoked nodulation in a time- and bacterial dose-related manner. Pharmaceutical inhibitors of PLA_2_, COX and LOX inhibited the nodulation response in dose-dependent ways and the inhibition was reversed by co-injecting AA with the inhibitor dexamethasone. *E. coli* challenge also stimulated PLA_2_ activity in hemolymph and in the combined hemocyte/fat body preparations. We identified five genes encoding sPLA_2_ by *in silico* interrogation of the *T. castaneum* genome, designated TcsPLA_2_A through E. To ensure these genes encode an active enzyme, we expressed each of the genes in competent *E. coli* cells, which yielded 4- to 6-fold increases in PLA_2_ activity. The activity of all five recombinant PLA_2_s was inhibited by *p*-bromophenacyl bromide, a specific sPLA_2_ inhibitor. We designed dsRNAs unique to mRNAs for each of the five genes. After injecting the dsRNAs into experimental larvae, we found gene-specific reduction or elimination of the corresponding mRNAs. In a final experiment, we injected the dsRNAs and *E. coli* into experimental larvae. At 4 h post-infection we recorded substantial and statistically significant reductions in nodulation for four of the five genes. These reductions in nodulation were significantly reversed by co-injecting AA with the dsRNA. Overall, this work identified four genes encoding PLA_2_s that act in the nodulation reactions to bacterial infections in *T. castaneum*. Separately silencing each of these genes inhibited nodulation reactions to infection for 1–3 days. Exogenous dsRNA constructs can be selectively expressed in plant tissues. We envision constructs that inhibit expression of PLA_2_ encoding genes in herbivorous pests will be taken in to the pests during feeding on crop plants. As shown in our proof-of-concept paper [51], silencing the PLA_2_s can compromise immune reactions in pests.

Developing new pest management technologies will contribute to global food security in a world with a rapidly growing human population, the fastest population growth in the history of our species. The research just described forms the conceptual basis for one novel approach: selectively crippling the ability of pest insects to respond to adventitious or applied infections and invasions at the field level. Many barriers remain to be identified and overcome before practical application of this [52] and other approaches [53] to new technologies can be achieved. These barriers also form a compelling urgency for research in insect science. 

## 9. Conclusions

It has been about 20 years since the first suggestion that PGs and other eicosanoids exert crucial actions in insect immune functions. Because these compounds act in many areas of human and veterinary pathophysiology, a very large industrial, government and university enterprise is focused on discovering subtle and selective tools to modulate eicosanoids. These tools include the classical aspirin and other non-steroidal anti-inflammatory drugs (NSAIDs) that act by inhibiting COX, the core enzyme in PG biosynthesis. Newer, more subtle NSAIDs are now on the market and more are in development. It is not a very long jump from this very successful research to the idea that eicosanoid-mediated immunity can, at least in theory, be manipulated in insects [78]. We have considered two approaches to manipulating insect immunity. A pharmacological approach, based on discovery of insect-specific COX inhibitors, has not yet been exploited. We have shown that cellular immune reactions to bacterial infection can be impaired in the model pest, *T. castaneum*, by deploying RNAi to silence genes encoding PLA2s [51].

Insect immunity can be seen as an “emergency” system, evolved to react to various emergency situations, including wounds, infections and invasions. The effects of crippling insect immune reactions to these situations appear rapidly. The design and deployment of immune-impairing tools is one of the sorely-needed new technologies of insect pest management. 
